# Tuberculosis of the Hip-Joint

**Published:** 1909-03-13

**Authors:** 


### Surgery.

### TUBERCULOSIS OF THE HIP-JOINT.

A tuberculous hip is, in its early stages, an insidious condition, and one that may easily be overlooked; and the consequences of such an oversight may be genuinely disastrous, since the prognosis is only good when the disease is recognised early and the trouble is efficiently treated from the very beginning.

As in tuberculosis of any joint the disease may start in either of the bones forming the articulation or in the synovial membrane. Unfortunately the primary lesion is usually situated in the bone; unfortunately because in this case caries of the articular surface occurs early, and the prognosis, even if efficient treatment be started early, is not so good as in the other. But if the condition be left untreated, all the joint structures will be successively involved wherever the disease started.

One reason why the disease is often overlooked in its early stages is that the condition is essentially one of infantile life: more than half of the cases occur before the age of puberty; and young children are unable to give a definite account of their symptoms. The first symptom is usually pain in the joint. After a short time the child is noticed to walk with a limp, by which means attention is called to its condition.

Any child presenting these two symptoms should be suspected of a tuberculous hip until it can be proved conclusively that there is no ground for the suspicion. Clinical examination may in the earliest stages reveal little or nothing; but there are two signs, each slight in themselves, which if found give grounds for regarding the case as being probably one of tuberculosis. One is a slight fulness of Scarpa's triangle compared with the sound side, and the other is pain if the joint is fully flexed or fully extended; in fact it may be impossible to perform either of these manipulations. An *x*-ray photograph should always be taken, but, however good the skiagram itself may be, it is possible that an early focus may give no evidence, and this will certainly be the case if the lesion is a synovial one. It may be of assistance to take the opsonic index of the patient to the tubercle bacillus, but it must be pointed out that a single observation is only misleading. The index must be estimated several times, and on each occasion on which the blood is taken for this purpose the temperature of the patient must be known. If it is found in a number of observations that the opsonic index varies inversely as the temperature, that is to say, that the index is low while the temperature is raised and *vice versa*, then there is presumptive evidence that the lesion is a tuberculous one.

In such a case the child should be kept absolutely at rest for a month or six weeks, at the seaside for preference, and then he should be examined again. If the signs persist a diagnosis of tuberculous hip may be confidently made. The same difficulty in diagnosis does not present itself in the later stages.

After a time flexion and abduction of the hip joint supervene. This happens because the joint is kept rigid by Nature in the position of greatest comfort, and this position is one of flexion and slight abduction. Thus is brought about contraction of the ilio-psoas muscle. Two things will occur if the child is allowed to walk about after the onset of this stage. He will unwittingly compensate for the deformity, and in order to walk erect, with his legs parallel, he will hide the flexion by lordosis of the vertebral column, and bring his legs parallel again by tilting the pelvis down towards the affected side.

In examining such a case these secondary changes must be borne in mind. When the child lies on his back there will be no apparent flexion, but if the sound hip be flexed until the knee is brought into the abdomen until the lordosis is eliminated (this can be known by keeping the other hand in the small of the back) it will be found that the knee on the affected side is raised from the bed.

Further, the tilting down of the pelvis will cause an apparent lengthening of the affected limb. That this is apparent and not real may be shown by taking careful measurements. But here one may be misled for want of a simple precaution. In measuring it must be seen that the pelvis is straight and that the two limbs are in the same position when measured, *i.e.* if the affected limb is flexed and abducted the sound limb must be put in the same position, measurements should be taken from the iliac spine to the tip of the internal malleolus on each side, the tape being held down at the inner side of the knee, halfway between the front and back of the limb. To prove the truth of this statement it is only necessary to take an ordinary individual and measure his legs, one being extended and abducted while the other is flexed and abducted, and an appreciable difference in the length on the two sides will be found.

Starting pains at night, which are characteristic of all tuberculous joint disease, afford evidence that the disease has advanced to the stage of attacking the articular surfaces. Sleep relaxes the periarticular muscles, and the eroded surfaces move on each other; the muscles contract to keep the surfaces at rest, and a further contact occurs which is painful and wakes the patient with a start.

In the later stages the deformity of the hip is different. There is now internal rotation and adduction of the limb. This is due not as has been suggested to tiring out of the ilio-psoas muscle but to the fact that the acetabulum is eroded, and the periarticular ligaments are weakened by the disease. As a direct result there is some degree of dorsal dislocation of the joint, with its typical deformity. Just as in the previous stage the patient compensates for the displacement, but in order to get his legs parallel he now tilts the pelvis *up* on the affected side, so that there is apparent shortening. Careful measurement, taken with due regard to the precautions already mentioned, will demonstrate that the shortening is not real. When this stage has been reached it may be regarded as certain that the interior of the joint is completely disorganised.

(*To be continued.*)

